# Gray and White Matter Voxel-Based Morphometry of Alzheimer’s Disease With and Without Significant Cerebrovascular Pathologies

**DOI:** 10.1177/26331055231225657

**Published:** 2024-01-31

**Authors:** Chandan Saha, Chase R Figley, Zeinab Dastgheib, Brian J Lithgow, Zahra Moussavi

**Affiliations:** 1 Biomedical Engineering Program, University of Manitoba, Winnipeg, MB, Canada; 2 Department of Radiology, University of Manitoba, Winnipeg, MB, Canada

**Keywords:** Alzheimer’s disease, AD with cerebrovascular disease, magnetic resonance imaging, voxel-based morphometry, gray matter, white matter

## Abstract

Alzheimer’s disease (AD) is the most common type of dementia, and AD individuals often present significant cerebrovascular disease (CVD) symptomology. AD with significant levels of CVD is frequently labeled mixed dementia (or sometimes AD-CVD), and the differentiation of these two neuropathologies (AD, AD-CVD) from each other is challenging, especially at early stages. In this study, we compared the gray matter (GM) and white matter (WM) volumes in AD (n = 83) and AD-CVD (n = 37) individuals compared with those of cognitively healthy controls (n = 85) using voxel-based morphometry (VBM) of their MRI scans. The control individuals, matched for age and sex with our two dementia groups, were taken from the ADNI. The VBM analysis showed widespread patterns of significantly lower GM and WM volume in both dementia groups compared to the control group (*P* < .05, family-wise error corrected). While comparing with AD-CVD, the AD group mainly demonstrated a trend of lower volumes in the GM of the left putamen and right hippocampus and WM of the right thalamus (uncorrected *P* < .005 with cluster threshold, *K* = 10). The AD-CVD group relative to AD tended to present lower GM and WM volumes, mainly in the cerebellar lobules and right brainstem regions, respectively (uncorrected *P* < .005 with cluster threshold, *K* = 10). Although finding a discriminatory feature in structural MRI data between AD and AD-CVD neuropathologies is challenging, these results provide preliminary evidence that demands further investigation in a larger sample size.

## Introduction

Dementia is a general medical condition that affects an individual's cognitive and behavioral abilities and has diverse subtypes, including Alzheimer’s disease (AD), vascular dementia (VaD), Parkinson’s dementia, frontotemporal dementia, Lewy body dementia, and so on.^1^ AD and VaD are common types of dementia, which comprise about 60 and 20% of total cases, respectively,^2^ and both have characterized pathology.^3^ In AD pathology, the deposition of amyloid plaques and tau tangles in the brain leads to neuronal synaptic loss and subsequently deteriorates cognitive functions.^4,5^ In contrast, VaD refers to the cognitive decline resulting from vascular issues, including ischemic strokes, hemorrhages, and hypoperfusion.^6^ Frequently, individuals diagnosed with AD present significant cerebrovascular disease (CVD) symptomology; herein, we labeled this group as AD-CVD.^3^ Due to the confounded pathology of AD-CVD and its overlapping symptoms with AD,^7^ the differential diagnosis of AD-CVD from AD individuals is challenging, especially at early stages and thus a focus of this study.

While there is no unified approach for the diagnosis of AD-CVD,^8^ one of the common approaches is utilizing the Hachinski Ischemic Score (HIS), a clinical scoring tool comprising 13 items,^9,10^ to separate AD-CVD from AD. In particular, the representative HIS score for AD-CVD is >4 but <7, while for AD, it is ⩽4.^11^ However, this rating scale alone offers limited accuracy^12^ and lacks taking advantage of CVD-related brain imaging data.^7,13^ Another approach to diagnose VaD and AD-CVD is the National Institute of Neurological Disorders and Stroke—the Association Internationale pour la Recherche et l’Enseignement en Neurosciences (NINDS-AIREN) scale^14^ that incorporates brain imaging outcomes.

Imaging biomarkers can play an important role in increasing the certainty of the dementia diagnosis.^15,16^ Depending on the modalities, imaging biomarkers trace structural, functional, and molecular changes in the brain.^16,17^ Magnetic resonance imaging (MRI) is a widely used tool that can assist in identifying dementia types and their underlying pathologies.^15^ Dementia causes regional atrophy in gray matter (GM) and white matter (WM) of the brain, which can be detected using structural MRI techniques.^18,19^ Previous studies reported patterns of this structural alteration in AD and VaD.^20-23^ However, to the best of our knowledge, this pattern in AD-CVD is not well characterized. For identifying dementia types, cerebrospinal fluid (CSF) markers (levels of amyloid beta 42 and tau protein) have been reported in past studies,^24,25^ but the CSF collection process is invasive, requiring lumbar puncture. The amyloid burden tracer called^11^C-Pittsburg compound-B positron emission tomography^26^ has been utilized in recent studies^27,28^; however, it is expensive and has limited use in clinical practice.^29^ Alternatively, analysis of structural MRI data in relation to AD and AD-CVD might provide additional information, such as any trend of atrophic patterns in GM or WM that would facilitate their differentiation.

In the context of AD-CVD, it may be challenging to characterize its differential atrophic patterns due to its diverse localization of brain lesions and co-occurred pathologies.^30^ Our previous pilot study^31^ regarding AD and AD-CVD separation executed a region of interest (ROI)-based analysis on a few selected vulnerable areas in brain MRI, such as ventricles, hippocampus, frontal gyrus and precuneus and estimated white matter hyperintensities (WMHs), a marker of CVD. In that study^31^ we did not notice a substantial distinction between AD and AD-CVD in those limited areas and WMHs, suggesting further analysis incorporating other additional brain regions in a larger sample. Herein, we conducted a whole-brain comparison in our two AD and AD-CVD groups and their age and sex-matched cognitively healthy controls. We aimed to explore whether the AD, AD-CVD, and controls differed in GM and WM while comparing them with each other and voxel-based morphometry (VBM) was applied herein. While previous studies on VBM^20,21^ widely focused on GM or WM volumetric atrophic patterns in AD people compared to controls, we explored these patterns in the AD-CVD group.

The novel contribution of this paper is to investigate the AD-CVD brains’ structural differences using their MRI scans for the first time in comparison to those in the AD population.

## Methodology

### Participants

Brain MRI data has been collected from 141 participants as part of a clinical trial studying the effects of repetitive transcranial magnetic stimulation (rTMS) on Alzheimer’s individuals in three locations (Winnipeg, Montreal, and Melbourne).^32^ We excluded 21 participants with insufficient image quality and significantly different MRI scanning metrics (echo time, inversion time, and repetition time). We utilized the modified HIS similar to our team’s previous study^13^ to group our remaining 120 participants as AD (n = 83) or AD-CVD (n = 37). Because of accuracy improvement in modified HIS, as reported in Molsa et al,^12^ the highest cut-off was ⩽3 for AD, and the score range for AD-CVD was ⩾4 but ⩽7. The Mean ± SD of modified HIS for the AD and AD-CVD groups were 1.3 ± 1.1 and 4.5 ± 1.1, respectively. Our classified AD-CVD subjects also fulfilled the definitions of “AD with CVD” following the NINDS-AIREN criteria.^14^ Additionally, we considered the clinical neuropsychiatrist or neurologist’s diagnosis utilizing patient history, imaging findings and cognitive assessments. All participants gave written consent prior to participation in the trial^33^ according to the Declaration of Helsinki, which was approved by the Biomedical Research Ethics Boards of all three study sites (Winnipeg, Montreal, and Melbourne).

Since our clinical trial of rTMS did not include any control participants, we downloaded brain MRI data of 85 age- and sex-matched cognitively normal controls from the ADNI database (http://adni.loni.usc.edu/) to compare our AD and AD-CVD participants. Notably, the ADNI study included subjects with their self-defined modified HIS ⩽ 4.^34^

### MRI acquisition

We used 3D T1-weighed magnetization prepared rapid acquisition gradient-echo (MPRAGE) scans for the AD, and AD-CVD participants, and all data were acquired using 3T Siemens MRI systems—specifically, a 3T Verio (Winnipeg), Prisma (Montreal), and Skyra (Melbourne). Scanning parameters were slightly different in each of the three locations; however, slice thickness, repetition time, echo time, inversion time, and flip angle were 0.9 to 1.2 mm, 1800 to 2300 milliseconds, 2.19 to 2.98 milliseconds, 900/1100 milliseconds, and 8 to 12°, respectively. We also downloaded 3T T1-weighed MPRAGE MRI data for the cognitively normal controls from the ADNI database. The MRI scanning parameters of the controls were: slice thickness = 1.2 mm, echo time = 3 milliseconds, repetition time = 2300 milliseconds, inversion time = 900 milliseconds, and flip angle = 9°. The detailed MRI acquisition procedures following the ADNI protocol can also be found here.^34^

### Voxel-based morphometry (VBM) analysis

We conducted a VBM analysis on the MRI data utilizing the CAT12 toolbox (http://www.neuro.uni-jena.de/cat/) within the SPM12 software (https://www.fil.ion.ucl.ac.uk/spm/).^35^ All T1-weighted MR images were passed through a spatially adaptive non-local means^36^ denoising filter (strength “medium”) before an initial automatic registration and segmentation into GM, WM, and cerebrospinal fluid (CSF) tissues, utilizing the “unified segmentation” method.^37^ This initial segmentation was further refined to reach the adaptive maximum a posteriori segmentation.^38^ Geodesic shooting registration^39^ was then applied to nonlinearly normalize the segmented tissues (GM and WM) to the Montreal Neurological Institute (MNI) template.^40^ The spatially normalized GM and WM tissue segmentations were modulated to preserve their relative volumes,^41^ and the modulated tissue images were then smoothed by a full width at half maximum (FWHM) Gaussian kernel [size: 8 × 8 × 8 mm^3^] before statistically evaluating the smoothed outputs. Finally, the automatic quality assurance metrics in each participant’s CAT12 report were manually checked to verify that the images had acceptable quality ratings. Moreover, prior studies reported the usage of a larger-sized smoothing kernel in the VBM analysis^42-44^; we smoothed the images using the FWHM Gaussian kernel of size 12 × 12 × 12 mm^3^ and presented the VBM results in the supplementary file for your interest.

### Statistical analysis

Statistical analysis of the subjects’ demographic and group characteristics (Table 1) was conducted using RStudio (ver. 1.4.1106) in the R platform.^45,46^ A two proportions test was considered for comparing male/female ratios between groups. Depending on the distribution of continuous data (i.e., age and other group characteristics), we ran either a parametric two-sample *t*-test (for normally distributed data) or a non-parametric Mann–Whitney *U*-test (for non-normally distributed data) to evaluate the statistical significance of each comparison across the three groups.

**Table 1.** Subjects’ demographic details and group characteristics.

	AD (*n* = 83) mean ± SD	AD-CVD (*n* = 37) mean ± SD	Controls (*n* = 85) mean ± SD	AD vs controls	AD-CVD vs controls	AD vs AD-CVD
Sex (M, F)^†^	44, 39	24, 13	47, 38	χ^2^ = 0.02, *P* = .89	χ^2^ = 0.62, *P* = .43	χ^2^ = 1.02, *P* = .31
Age (years)‡	73.1 ± 8.0	76.0 ± 7.8	74.4 ± 6.4	*t* = −1.12, *P* = 0.26	*t* = 1.10, *P* = .27	*t* = −1.84, *P* = .07
MoCA††	15.3 ± 5.1	16.5 ± 5.1	26.3 ± 2.5	*W* = 189.5, *P* < .001	*W* = 107, *P* < .001	*W* = 1339.5, *P* = .27
ADAS-Cog††	24.5 ± 8.6	22.0 ± 9.1	6.0 ± 3.0	*W* = 6955.5, *P* < .001	*W* = 2953.5, *P* < .001	*W* = 1741, *P* = .15
CDR††	1.1 ± 0.3	1.1 ± 0.3	0.0 ± 0.0	*W* = 7055, *P* < .001	*W* = 3145, *P* < .001	*W* = 1515.5, *P* = .86

Abbreviations: AD, Alzheimer’s disease; ADAS-Cog: Alzheimer’s disease assessment scale-cognitive subscale; CDR, clinical dementia rating; AD-CVD, AD with cerebrovascular disease; MoCA, montreal cognitive assessment; SD, standard deviation.

In three group comparisons, statistically significant difference was only considered, if *P* ⩽ .017 (α = .05/3, Bonferroni correction).

† Two-proportions test.

‡ Two-sample *t*-test.

†† Mann–Whitney *U* test or Wilcoxon rank-sum test.

The hypotheses were: a) the AD-CVD group has substantial GM and WM differences compared to those in controls, and b) the AD and AD-CVD groups would present a volumetric difference in VBM.

Group differences in normalized, modulated, and smoothed GM and WM images were separately assessed using a general linear model in SPM12. A full factorial design with three levels (AD, AD-CVD, controls) was performed with the inclusion of age, sex, total intracranial volume (TIV), and MRI location (Winnipeg, Montreal, Melbourne, and ADNI) as covariates in the design matrix. The following t-contrasts were investigated to compare between groups: AD < controls, AD-CVD < controls, and AD < AD-CVD, representing lower volume in AD than in controls, lower volume in AD-CVD than in controls, and lower volume in AD than in AD-CVD, respectively (as well as the opposite t-contrasts: i.e., AD > controls, AD-CVD > controls, and AD > AD-CVD). The family-wise error (FWE) was corrected in the model at *P* < .05. A liberal threshold with uncorrected *P* < .005 and cluster extent, *K* = 10, has been reported in Lieberman and Cunningham^47^ and taken into consideration in this study if no regions with significant differences were found after multiple comparison corrections. It is worth mentioning that the peak MNI coordinate regions from the VBM analysis, reported in the following results section, were found using the XjView toolbox (https://www.alivelearn.net/xjview/).

## Results

### Group comparisons

Table 1 details the subjects’ demographic and cognitive data. From the two-proportions tests for sex and two-sample *t*-test for age, no statistical differences were observed between the AD, AD-CVD and control groups. As expected, the control individuals had significantly higher MoCA and lower ADAS-Cog and CDR scores than the dementia groups (*P* < .001); however, no statistical differences were found across these scores for AD and AD-CVD groups.

### Gray matter VBM

**Figure 1.**
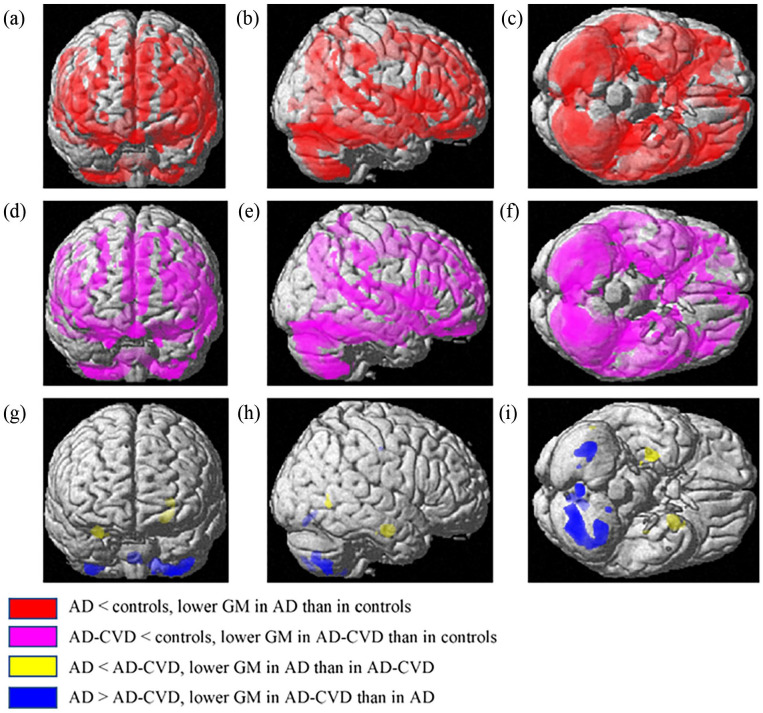
Anatomical rendering in SPM displays VBM results on gray matter (GM). The left, middle, and right columns are anterior, right, and bottom views of the 3D brain template, respectively. In the t-contrasts of AD < controls (a-c) and AD-CVD < controls (d-f), the family-wise error (FWE) was corrected at *P* = .05; an uncorrected *P* < .005 with *K* = 10^47^ was used for the AD < AD-CVD and AD > AD-CVD contrasts (g-i). Both dementia groups demonstrated widespread patterns of significantly lower GM volume than controls, and the GM volumetric difference patterns of AD and AD-CVD compared to controls appeared to be overlapped. In comparison between the two dementia groups, AD showed a trend of lower GM, particularly in the left putamen and right hippocampus compared to AD-CVD. Relative to AD, the AD-CVD had lower GM, mainly in the lobules of the cerebellum and vermis. Abbreviations: AD, Alzheimer’s disease; AD-CVD, AD with cerebrovascular disease.

Compared to controls, the AD group had significantly lower GM volume in several brain regions after correcting for age, sex, TIV, and MRI location (Table 2 and Figure 1a-c). The largest of these clusters (216 810 voxels) was primarily located in the right inferior temporal gyrus and right parahippocampal gyrus but also extended to other brain areas. The second largest cluster (2921 voxels) was mostly centered in the left thalamus, and the third largest cluster (1528 voxels) was in the bilateral gyrus rectus and the orbital part of the right superior frontal gyrus. Another large cluster (1096 voxels) was observed in the dorsolateral part of the left superior frontal gyrus and left precentral gyrus. Other significant clusters were also found in the right angular gyrus and right superior occipital gyrus, right precentral gyrus, the temporal pole of the right superior temporal gyrus, orbital part of the right superior frontal gyrus, right thalamus, and left anterior cingulate and paracingulate gyri. Other clusters with a size of <100 voxels were found in brain regions, as detailed in Table 2. No substantial differences were found for the opposite *t*-contrast (AD > controls).

**Table 2.** Regions of lower gray matter (GM) volume in AD compared with controls (*t* contrast: AD < controls).

Voxel level P_FWE-corr_	Extent	*t* Values of voxel-level	Peak MNI coordinates (mm)			Side (L: Left, R: Right)	Regions
			x	y	z		
<0.001	216 810	14.3	48	−42	−26	R	Inferior temporal gyrus
<0.001		13.1	22	−10	−30	R	Parahippocampal gyrus
<0.001		13.1	18	0	−14	R	Parahippocampal gyrus
<0.001	71	11.1	−30	−4	−44	L	Inferior temporal gyrus
<0.001	1	10.9	−22	2	−32	L	Parahippocampal gyrus
<0.001	2921	9.2	−2	−6	4	L	Thalamus
<0.001		8.9	−4	−14	4	L	Thalamus
<0.001	61	8.7	−14	−34	2	L	Thalamus
<0.001	1096	8.2	−22	2	60	L	Superior frontal gyrus, dorsolateral
<0.001		6.4	−24	−12	64	L	Precentral gyrus
<0.001		6.0	−32	0	56	L	Precentral gyrus
<0.001	156	7.9	54	8	−8	R	Temporal pole: superior temporal gyrus
<0.001	3	7.3	−58	−56	−12	L	Inferior temporal gyrus
<0.001	1528	7.1	−2	54	−24	L	Gyrus rectus
<0.001		6.5	4	44	−24	R	Gyrus rectus
<0.001		6.1	10	50	−22	R	Superior frontal gyrus, orbital part
<0.001	60	7.1	60	−6	−12	R	Middle temporal gyrus
<0.001	23	7.0	58	−2	−16	R	Middle temporal gyrus
<0.001	142	6.9	16	−34	2	R	Thalamus
<0.001	3	6.6	62	−42	−10	R	Inferior temporal gyrus
<0.001	513	6.5	38	−66	42	R	Angular gyrus
0.001		5.5	26	−66	48	R	Superior occipital gyrus
0.002		5.2	46	−64	36	R	Angular gyrus
<0.001	13	6.2	−22	−64	54	L	Superior parietal gyrus
< 0.001	175	5.7	44	2	44	R	Precentral gyrus
< 0.001	14	5.5	60	0	−2	R	Superior temporal gyrus
0.001	143	5.3	28	62	−4	R	Superior frontal gyrus, orbital part
0.001	61	5.3	16	52	34	R	Superior frontal gyrus, dorsolateral
0.002	2	5.2	−4	−62	56	L	Precuneus
0.008	102	4.9	−4	52	−2	L	Anterior cingulate and paracingulate gyri
0.009	7	4.8	12	56	24	R	Superior frontal gyrus, medial
0.013	9	4.7	38	−46	54	R	Inferior parietal gyrus
0.014	10	4.7	30	−52	60	R	Superior parietal gyrus
0.021	18	4.6	−14	56	−16	L	Middle frontal gyrus, orbital part
0.021	14	4.6	34	−74	32	R	Middle occipital gyrus
0.027	2	4.5	−60	−32	4	L	Middle temporal gyrus
0.029	10	4.5	−58	−34	34	L	Supramarginal gyrus
0.036	1	4.5	−58	−36	36	L	Supramarginal gyrus
0.037	7	4.5	36	54	−10	R	Middle frontal gyrus, orbital part
0.040	3	4.4	32	54	−2	R	Middle frontal gyrus, orbital part
0.043	1	4.4	20	−40	66	R	Postcentral gyrus
0.044	4	4.4	−6	52	6	L	Superior frontal gyrus, medial
0.046	1	4.4	60	−18	28	R	Supramarginal gyrus
0.049	1	4.4	−34	14	−10	L	Insula

Abbreviations: MNI, montreal neurological institute; P_FWE-corr_, family-wise error (FWE) corrected *P*-values.

The AD-CVD individuals also had substantially lower GM volume in brain regions relative to the control group, as described in Table 3 and Figure 1d to f. Similar to the AD group, the largest of these clusters (183 821 voxels) was primarily positioned in the right inferior temporal gyrus and parahippocampal gyrus and covered several brain regions of AD-CVD. The second largest cluster with 2072 voxels was mainly centered in the left thalamus; the third largest cluster (1756 voxels) was in the bilateral gyrus rectus and orbital part of the right superior frontal gyrus, similar to the AD group. Large clusters of GM differences in AD-CVD compared to controls were also found in regions of the dorsolateral part of the left superior frontal gyrus and left precentral gyrus, left superior occipital gyrus, left parts of the postcentral gyrus, supramarginal gyrus, and inferior parietal gyrus, right precentral gyrus and middle frontal gyrus, right inferior parietal gyrus, the temporal pole of the right superior temporal gyrus, and lobule 2 of left cerebellum curs. Other clusters with <100 voxels were found in different brain regions, as shown in Table 3. No significant differences were noticed for the opposite t-contrast (AD-CVD > controls).

**Table 3.** Regions of lower gray matter (GM) volume in AD-CVD compared with controls (*t* contrast: AD-CVD < controls).

Voxel level P_FWE-corr_	Extent	*t* Values of voxel-level	Peak MNI coordinates (mm)			Side (L: Left, R: Right)	Regions
			*x*	*y*	*z*		
<0.001	183 821	12.9	48	−42	−26	R	Inferior temporal gyrus
<0.001		11.6	20	−8	−32	R	Parahippocampal gyrus
<0.001		11.3	20	2	−34	R	Parahippocampal gyrus
<0.001	71	10.3	−30	−4	−46	L	Fusiform gyrus
<0.001	1	9.6	−22	2	−32	L	Parahippocampal gyrus
<0.001	2072	8.2	−2	−6	2	L	Thalamus
<0.001		7.8	−4	−12	10	L	Thalamus
<0.001		7.5	−4	−14	2	L	Thalamus
<0.001	1756	7.6	−2	52	−24	L	Gyrus rectus
<0.001		6.9	4	44	−24	R	Gyrus rectus
<0.001		6.1	10	50	−22	R	Superior frontal gyrus, orbital part
<0.001	144	7.6	54	8	−8	R	Temporal pole: superior temporal gyrus
<0.001	836	7.2	−22	2	60	L	Superior frontal gyrus, dorsolateral
<0.001		5.6	−34	0	54	L	Precentral gyrus
<0.001	61	7.2	−14	−34	2	L	Thalamus
<0.001	3	6.4	62	−42	−10	R	Inferior temporal gyrus
<0.001	3	6.3	−58	−56	−12	L	Inferior temporal gyrus
<0.001	60	6.2	62	−10	−10	R	Superior temporal gyrus
<0.001	624	6.1	−42	−32	46	L	Postcentral gyrus
<0.001		5.7	−54	−44	30	L	Supramarginal gyrus
<0.001		5.5	−46	−40	48	L	Inferior parietal gyrus
<0.001	416	6.0	26	−8	56	R	Precentral gyrus
0.043		4.4	36	−2	54	R	Middle frontal gyrus
<0.001	170	5.6	44	−40	50	R	Inferior parietal gyrus
<0.001	23	5.6	58	−2	−16	R	Middle temporal gyrus
<0.001	14	5.5	60	−2	−2	R	Superior temporal gyrus
0.001	799	5.3	−26	−82	30	L	Superior occipital gyrus
0.004		5.0	−24	−76	42	L	Superior occipital gyrus
0.002	128	5.2	44	2	44	R	Precentral gyrus
0.002	131	5.2	−14	−88	−28	L	Cerebellum crus (2)
0.006	18	4.9	16	−34	2	R	Thalamus
0.007	38	4.9	−60	−48	−12	L	Middle temporal gyrus
0.012	31	4.8	−46	−36	20	L	Superior temporal gyrus
0.018	3	4.7	16	−40	0	R	Precuneus
0.020	52	4.6	4	−48	−40	R	Cerebellum (9)
0.022	14	4.6	16	52	32	R	Superior frontal gyrus, dorsolateral
0.029	10	4.5	60	−18	28	R	Supramarginal gyrus
0.035	4	4.5	4	−92	−2	R	Calcarine fissure and surrounding cortex
0.039	3	4.4	−20	−86	−32	L	Cerebellum crus (2)
0.042	2	4.4	50	−14	16	R	Rolandic operculum
0.045	1	4.4	38	−64	46	R	Angular gyrus

Abbreviations: MNI, montreal neurological institute; P_FWE-corr_, Family-wise error (FWE) corrected *P*-values.

Amongst the contrasts between the two dementia groups (AD < AD-CVD, AD > AD-CVD), a significant difference was not observed in VBM analysis using P_FWE-corr_ < 0.05. However, using an uncorrected threshold of *P* < .005 with *K* = 10, as presented in Table 4 and Figure 1g to i, AD subjects exhibited a trend of lower GM volume in the left putamen, right hippocampus, left insula, right middle temporal gyrus, and parahippocampal gyrus compared to AD-CVD. In the opposite contrast of AD > AD-CVD, AD-CVD showed a trend of lower GM volume in the left lobules (6, 7b, 8, and 9) and right lobules (8) of the cerebellar hemisphere, lobule 8 of the right vermis, and right middle cingulate gyrus relative to AD.

**Table 4.** Regions of gray matter (GM) and white matter (WM) difference in AD compared to AD-CVD.

*t*-Contrast	Extent	*t* Values of voxel-level	Peak MNI			Side (L: Left, R: Right)	Regions
			coordinates (mm)				
			*x*	*y*	*z*		
GM							
AD < AD-CVD	893	3.4	−24	4	−12	L	Putamen
		2.8	−26	16	0	L	Putamen
		2.8	−28	8	2	L	Putamen
	595	3.3	32	−8	−22	R	Hippocampus
	42	2.9	−32	−16	4	L	Insula
	85	2.9	52	−60	2	R	Middle temporal gyrus
	11	2.7	22	−20	−26	R	Parahippocampal gyrus
AD > AD-CVD	4464	4.2	−28	−76	−52	L	Cerebellum (7b)
		3.6	−34	−52	−58	L	Cerebellum (8)
		3.4	−40	−68	−50	L	Cerebellum crus (2)
	1233	3.7	30	−64	−48	R	Cerebellum (8)
		3.1	36	−60	−58	R	Cerebellum (8)
	518	3.4	2	−70	−46	R	Vermis (8)
	653	3.2	−10	−66	−8	L	Cerebellum (6)
		3.2	−4	−76	−14	L	Cerebellum (6)
	41	3.1	−14	−46	−58	L	Cerebellum (9)
	12	2.7	10	−16	44	R	Middle cingulate gyrus
	14	2.7	22	−70	−48	R	Cerebellum (8)
WM							
AD < AD-CVD	207	3.8	18	−16	16	R	Thalamus
	50	3.3	30	−12	−8	R	Putamen
	52	2.9	38	46	2	R	Middle frontal gyrus
AD > AD-CVD	1534	3.5	4	−36	−30	R	Brainstem
	13	2.9	12	−38	68	R	Postcentral gyrus
	15	2.9	8	−6	58	R	Supplementary motor area

Abbreviations: MNI, montreal neurological institute.

Results are presented here at a liberal threshold of uncorrected *P* < .005 and *K* = 10. AD and AD-CVD groups showed a tendency of GM and WM differences in the regions mentioned above while comparing them to each other.

### White matter VBM

**Figure 2.**
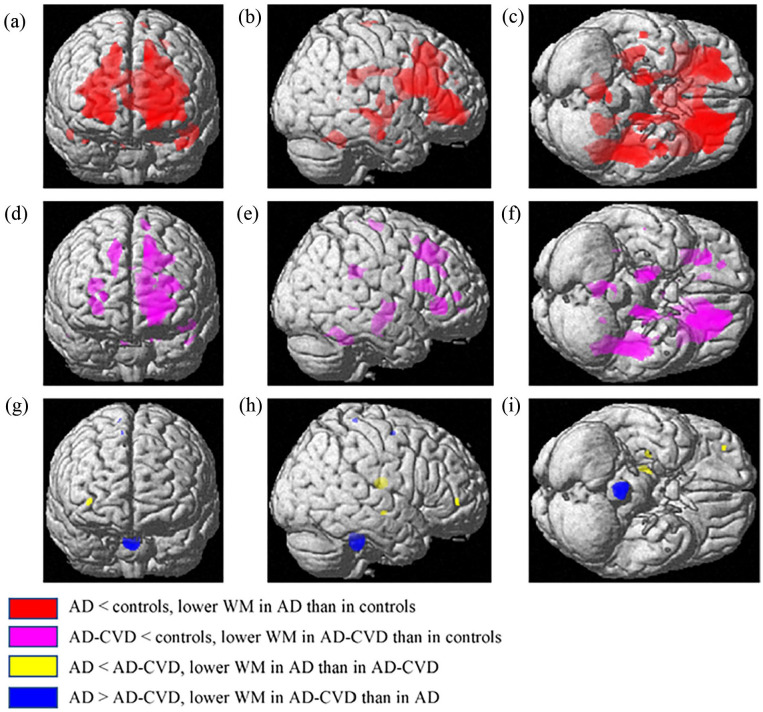
Anatomical rendering in SPM displays VBM results on white matter (WM). The left, middle, and right columns are anterior, right, and bottom views of the 3D brain template, respectively. In the t-contrasts of AD < controls (a-c) and AD-CVD < controls (d-f), the family-wise error (FWE) was corrected at *P* = .05 and an uncorrected thresholding of *P* < .005 with *K* = 10 was used for the AD < AD-CVD and AD > AD-CVD contrasts (g-i). Compared to controls, AD and AD-CVD groups presented a significantly lower WM volume in diverse brain areas, and most of the different regions appeared to be overlapped in both disease states. While comparing the two dementia groups, there was a subtle trend of WM difference in colored regions between AD and AD-CVD. Abbreviations: AD, Alzheimer’s disease; AD-CVD, AD with cerebrovascular disease.

Relative to the control group, AD individuals were found to have lower WM volume in several regions after correcting for age, sex, TIV, and MRI location (Table 5 and Figure 2a-c). These were mainly positioned in the left sides of the insula, middle cingulate gyrus, and sub-gyral regions, with the largest cluster of 82 442 voxels. The second largest cluster, with a size of 28 511 voxels, was primarily in the left middle temporal gyrus and left hippocampus; the third cluster (2281 voxels) was mainly noticed in the parahippocampal gyrus and lenticular nucleus of the left side of the brain. The right lobule (6) of the vermis and right cerebellar lobules (4, 5, 6), right hippocampus, left cerebellar lobules (4, 5, 6), and right inferior temporal gyrus presented a substantially lower WM in AD than in controls. Small clusters of <100 voxels were also found, as shown in Table 5 and Figure 2a to c. No substantial differences were found for the opposite *t*-contrast (AD > controls).

**Table 5.** Regions of lower white matter (WM) volume in AD compared with controls (*t* contrast: AD < controls).

Voxel level P_FWE-corr_	Extent	*t* Values of voxel-level	Peak MNI coordinates (mm)			Side (L: Left, R: Right)	Regions
			*x*	*y*	*z*		
<0.001	82 442	7.1	−30	20	14	L	Insula
<0.001		6.7	−10	16	36	L	Middle cingulate gyrus
<0.001		6.5	−22	46	−10	L	Sub-gyral
<0.001	28 511	6.7	−38	−20	−14	L	Middle temporal gyrus
<0.001		6.3	−34	−26	−8	L	Hippocampus
<0.001		6.2	−44	−56	0	L	Middle temporal gyrus
<0.001	2281	5.6	−16	−22	−18	L	Parahippocampal gyrus
0.001		5.2	−22	−16	4	L	Lentiform nucleus
0.002		5.1	−16	−16	−14	L	Parahippocampal gyrus
<0.001	652	5.4	−20	−52	−26	L	Cerebellum (6)
0.013		4.6	−20	−40	−26	L	Cerebellum (4, 5)
0.001	1324	5.2	6	−60	−24	R	Vermis (6)
0.001		5.1	4	−52	−18	R	Cerebellum (4, 5)
0.028		4.3	18	−52	−24	R	Cerebellum (6)
0.002	1239	5.1	36	−22	−8	R	Hippocampus
0.003		4.9	28	−28	−4	R	Hippocampus
0.004		4.8	34	−38	−2	R	Hippocampus
0.005	98	4.8	2	−44	−68	R	Brainstem
0.006	212	4.8	54	−16	−22	R	Inferior temporal gyrus
0.007	85	4.7	−10	−26	70	L	Paracentral lobule
0.014	30	4.5	−18	−46	6	L	Posterior cingulate gyrus
0.019	39	4.5	38	0	−26	R	Sub-gyral
0.026	21	4.4	10	−22	72	R	Paracentral lobule
0.028	3	4.4	24	−34	6	R	Hippocampus
0.029	21	4.3	12	4	42	R	Middle cingulate gyrus
0.037	18	4.3	−10	42	30	L	Superior frontal gyrus, medial
0.040	1	4.3	22	−26	−8	R	Hippocampus
0.040	12	4.2	−36	−64	20	L	Middle occipital gyrus
0.044	3	4.2	30	−50	8	R	Precuneus
0.047	1	4.2	−16	40	30	L	Superior frontal gyrus, dorsolateral

Abbreviations: MNI, montreal neurological institute; P_FWE-corr_, family-wise error (FWE) corrected *P*-values.

Comparing the AD-CVD and control groups (Table 6 and Figure 2d-f) revealed that the AD-CVD group showed substantially lower WM volumes in several regions, particularly in the left superior frontal gyrus (dorsolateral part), putamen, and cingulate gyrus, with the largest cluster of 22 973 voxels. The next largest cluster (5731 voxels) was mainly demonstrated in regions of the left brain's middle temporal gyrus and hippocampus. The surrounding areas of the right brain side, including the insula and middle frontal gyrus, thalamus and lenticular nucleus, dorsolateral and medial parts of the superior frontal gyrus and cingulate gyri indicated substantially lower WM in AD-CVD than in controls. Besides, relative to controls, the AD-CVD group demonstrated a lower WM in the left parahippocampal gyrus, bilateral cingulate gyrus, right lobules (4, 5, 6) of the vermis and left paracentral lobule. Further, as shown in Table 6 and Figure 2d to f, small clusters (>100 voxels) were noticed. No significant differences were noticed for the opposite t-contrast (AD-CVD > controls).

**Table 6.** Regions of lower white matter (WM) volume in AD-CVD compared with controls (*t* contrast: AD-CVD < controls).

Voxel level P_FWE-corr_	Extent	*t* Values of voxel-level	Peak MNI coordinates (mm)			Side (L: Left, R: Right)	Regions
			*x*	*y*	*z*		
<0.001	22 973	6.1	−12	22	46	L	Superior frontal gyrus, dorsolateral
<0.001		5.8	−20	20	−4	L	Putamen
<0.001		5.7	−12	16	36	L	Cingulate gyrus
<0.001	5731	5.6	−38	−18	−14	L	Middle temporal gyrus
0.001		5.3	−38	−26	−10	L	Hippocampus
0.001		5.3	−36	−34	−8	L	Hippocampus
<0.001	1101	5.6	6	−46	−24	R	Vermis (4, 5)
0.002		5.0	4	−58	−22	R	Vermis (6)
<0.001	2051	5.4	16	−16	−12	R	Thalamus
0.025		4.4	22	−14	2	R	Lenticular nucleus, pallidum
0.001	1969	5.2	−16	−22	−20	L	Parahippocampal gyrus
0.002		5.0	−16	−16	−12	L	Midbrain
0.010		4.6	−22	−16	4	L	Lentiform nucleus
0.003	1705	4.9	−6	−36	26	L	Cingulate gyrus
0.003		4.9	−4	−28	30	L	Cingulate gyrus
0.004		4.8	−10	−20	28	L	Cingulate gyrus
0.004	365	4.8	−10	−22	68	L	Paracentral lobule
0.005	1833	4.8	10	26	42	R	Superior frontal gyrus, medial
0.020		4.4	14	34	26	R	Anterior cingulate and paracingulate gyri
0.040		4.2	14	22	54	R	Superior frontal gyrus, dorsolateral
0.005	871	4.8	10	−38	28	R	Cingulate gyrus
0.008	14	4.7	34	−40	−2	R	Lateral ventricle
0.009	78	4.7	−48	−14	−28	L	Inferior temporal gyrus
0.009	2241	4.6	34	32	4	R	Insula
0.011		4.6	32	16	14	R	Insula
0.014		4.5	30	34	−4	R	Middle frontal gyrus
0.015	208	4.5	24	48	8	R	Superior frontal gyrus, dorsolateral
0.017	20	4.5	−28	−58	6	L	Lateral ventricle
0.024	31	4.4	−20	−50	−26	L	Cerebellum (6)
0.024	319	4.4	−20	−44	38	L	Sub-gyral
0.025	73	4.4	−16	46	20	L	Medial frontal gyrus
0.029	134	4.3	−14	−10	48	L	Cingulate gyrus
0.030	27	4.3	−2	−2	32	L	Middle cingulate gyrus
0.030	19	4.3	−16	4	−2	L	Lenticular nucleus, pallidum
0.041	1	4.2	−30	−56	6	L	Lateral ventricle
0.041	2	4.2	−2	−40	−24	L	Mid brain
0.042	3	4.2	52	−16	−24	R	Inferior temporal gyrus
0.043	4	4.2	−10	12	22	L	Lateral ventricle
0.047	1	4.2	2	−4	32	R	Middle cingulate gyrus
0.050	1	4.2	12	−38	68	R	Postcentral gyrus
0.050	1	4.2	−10	20	16	L	Corpus callosum

Abbreviations: MNI, montreal neurological institute; P_FWE-corr_, family-wise error (FWE) corrected *P*-values.

In comparing AD and AD-CVD using the uncorrected thresholding of *P* < .005 and *K* = 10, as shown in Table 4 and Figure 2g to i, AD tended to exhibit lower WM volumes in the right sides of the thalamus, putamen, and middle frontal gyrus than in AD-CVD. The AD-CVD appeared to demonstrate lower WM, mainly in regions of the right brainstem, compared to AD.

## Discussion

In this study, we applied VBM to explore the trend of possible differences in GM and WM of AD, AD-CVD and age and sex-matched controls. Both dementia groups (AD, AD-CVD) relative to controls presented an overall lower GM and WM volume in the brain.

Relative to controls, the regions with the most significant cluster of GM difference in both disorders (AD, AD-CVD) were mainly associated with the right sides of the inferior temporal gyrus and parahippocampal gyrus. The inferior temporal gyrus is involved in verbal fluency, and the impairments in these functions are early markers of AD.^48,49^ The parahippocampus encloses the hippocampus and contributes to the encoding and retrieval of memory.^50^ AD and AD-CVD groups also demonstrated a substantially lower GM surrounding the regions of the left thalamus, bilateral gyrus rectus, and parts of the superior frontal gyrus. Other cortical and deep subcortical structures also showed a significantly different GM volume in both disorders compared to controls. It is common to observe a widespread pattern of GM difference in AD individuals compared to controls;^51^ a previous meta-analysis on VBM reported GM changes at 334 coordinates in people with AD.^43^ Furthermore, the VBM on WM revealed that both dementia groups (AD, AD-CVD), relative to the control group, had WM differences in diverse brain areas. Nineteen different-sized clusters of WM differences in AD compared to controls were shown in VBM analysis covering a large area in the brain, whereas 27 clusters with varying sizes were shown in AD-CVD. Previous studies.^21,52-54^ on AD or cognitive impairment individuals showed the WM vulnerability of many of the regions that are reported in this study. In either GM or WM, the patterns of volumetric differences in AD and AD-CVD than in the control group overlap in most regions. It suggests that both dementia groups, despite their different levels of CVD, might have common pathologies of neurodegeneration, such as amyloid plaque and tau deposition.

In our comparison of two groups of dementia, VBM did not present a significant distinction between AD and AD-CVD in GM using thresholding of *P* < .05 for FWE correction. Our small sample sizes might limit the power to detect substantial volumetric discrepancies in the direct comparison of AD and AD-CVD. Indeed, the FWE correcting threshold of p is suggested in neuroimaging research;^55^ however, it appears overly conservative in whole-brain analysis,^56^ notably when AD and AD-CVD had many commonalities in symptoms and might also have subtle discrepancies to detect. Instead, a liberal threshold, uncorrected *P* < .005 with *K* = 10, reported in Lieberman and Cunningham^47^ for balancing type I and II errors, had been used in this study to find a possible trend of differences between AD and AD-CVD. Using this criterion, our AD tended to have lower GM around the regions of the left putamen and right hippocampus than AD-CVD. Previous studies^57,58^ on AD and VaD individuals reported that hippocampal atrophy is more severe in AD than in VaD. Another study^59^ also showed that the degenerative mechanism early impacts the hippocampus and putamen in the process of Alzheimer’s.

Another finding of this study was to observe the tendency of lower GM of the AD-CVD group, particularly in the cerebellar regions compared to the AD group (Table 3). The cerebellar lobules (6, 7b, 8) that showed the GM difference in this study are from the cerebellum’s posterior lobules, engaged in cognitive and sensorimotor tasks.^60^ Given that our AD-CVD group (mean age = 76, 2 out of 37 subjects < 65 years) was relatively older than the AD group (mean age = 73.1, 16 out of 83 subjects < 65 years) as well as aging alone could lessen the Purkinje cells of the cerebellum and its volume, reported in an animal study.^61^ We thus postulated that there might have been an age effect on our AD-CVD subjects for having lower GM in the cerebellar regions compared to AD.

In the WM analysis to compare two dementia groups, we observed a WM difference in AD compared to AD-CVD, mainly in the right thalamus. In contrast, one large cluster (1534 voxels) of difference was shown in the right brainstem of the AD-CVD compared to AD. The regions, that is, cerebellum and brainstem, that respectively showed lower GM and WM in AD-CVD participants compared to AD are known to be associated with the control of balance. It is noteworthy that the incidence of falls was reported to be higher, albeit not significantly, in VaD compared to AD.^62^ This might indicate the contribution of CVD by affecting the brain regions that are involved in the regulation of balance. Another finding is that the trend of differences between AD and AD-CVD was found to be evident in VBM analysis on GM rather than WM.

The supplementary file presented VBM results for comparing dementia groups (AD, AD-CVD) and controls (Supplemental Tables S1-S3, Supplemental Figures S1-S2) after smoothing the modulated GM and WM images by 12 mm FWHM Gaussian kernel. There were differences in VBM results of GM and WM between using 8 and 12 mm smoothing kernels for comparing AD versus controls and AD-CVD versus controls; in particular, these VBM results differed in the size and number of clusters of differences and the locations of peak MNI coordinates. Nevertheless, two different smoothing kernels did not considerably affect the VBM results in comparing AD and AD-CVD groups; these two VBM results seemed similar in most distinct areas (see Table 4 and Supplemental Table S2).

A few studies using other MRI sequences showed a potential abnormality in the WM fiber of the AD-CVD cohort. For example, Jang et al,^27^ using diffusion tensor imaging (DTI), reported a more extensive change of WM integrity in subcortical VaD and AD-CVD groups; however, the alternation was minimal in the pure AD group, compared to controls. Ji et al^30^ hypothesized that the AD-CVD subjects might have higher free water values, representing more disruption in WM than the AD without CVD. A recent pilot study Lee et al^63^ reported the potential abnormalities in WM tissue in the AD-CVD cohort.

This study has strengths as well as limitations, which suggest future works. 3T MRI data for all three groups were used in this study. VBM, an unbiased, automatic, and widely used tool for group-wise volumetric comparisons,^64^ reduced any potential bias associated with imaging analysis. We had diagnostic reports with brain imaging findings for 69 participants from their neurologist or neuropsychiatric; for the rest, we estimated the HIS scores based on the available information regarding their medication, medical history, and caregiver comments for grouping them as AD and AD-CVD. We also acknowledged our small sample size in this study. Nevertheless, the brain areas that showed a trend of potential differences between AD and AD-CVD encouraged conducting further analysis on large samples; they might also be informative in future studies for generating hypotheses in specific regions of interest for separating AD and AD-CVD.

## Conclusion

VBM exhibited a significant volumetric difference in GM and WM of AD and AD-CVD groups compared to control subjects. AD and AD-CVD also showed a trend of possible differences in GM and WM, and these preliminary findings demand further analysis in a larger population.

## Supplemental Material

sj-docx-1-exn-10.1177_26331055231225657 – Supplemental material for Gray and White Matter Voxel-Based Morphometry of Alzheimer’s Disease With and Without Significant Cerebrovascular Pathologies Click here for additional data file. Supplemental material, sj-docx-1-exn-10.1177_26331055231225657 for Gray and White Matter Voxel-Based Morphometry of Alzheimer’s Disease With and Without Significant Cerebrovascular Pathologies by Chandan Saha, Chase R Figley, Zeinab Dastgheib, Brian J Lithgow and Zahra Moussavi in Neuroscience Insights

## References

[1] Gouras GK . Dementia. In: Caplan MJ , ed. Reference Module in Biomedical Sciences. Elsevier; 2014.

[2] Rizzi L Rosset I Roriz-Cruz M . Global epidemiology of dementia: Alzheimer’s and vascular types. Biomed Res Int. 2014;2014:1-8. 10.1155/2014/908915 PMC4095986 25089278

[3] Ravona-Springer R Davidson M Noy S . Is the distinction between Alzheimer’s disease and vascular dementia possible and relevant? Dialogues Clin Neurosci. 2003;5:7-15. 22033677 10.31887/DCNS.2003.5.1/rravonaspringer PMC3181710

[4] Munoz DG Feldman H . Causes of Alzheimer’s disease. CMAJ. 2000;162:65. 11216203 PMC1232234

[5] Rajmohan R Reddy PH . Amyloid-beta and phosphorylated tau accumulations cause abnormalities at synapses of Alzheimer’s disease neurons. J Alzheimer’s Dis. 2017;57:975-999. 27567878 10.3233/JAD-160612 PMC5793225

[6] Vascular dementia: causes, symptoms, and treatments. National Institute on Aging. Accessed July 3, 2022. https://www.nia.nih.gov/health/vascular-dementia

[7] Dastgheib ZA Lithgow BJ Moussavi ZK . An unbiased algorithm for objective separation of Alzheimer’s, Alzheimer’s mixed with cerebrovascular symptomology, and healthy controls from one another using electrovestibulography (EVestG). Med Biol Eng Comput. 2022;60:797-810. 35102489 10.1007/s11517-022-02507-1

[8] Custodio N Montesinos R Lira D Herrera-Pérez E Bardales Y Valeriano-Lorenzo L . Mixed dementia: a review of the evidence. Dement Neuropsychol. 2017;11:364. 29354216 10.1590/1980-57642016dn11-040005 PMC5769994

[9] Hachinski VC Iliff LD Zilhka E , et al. Cerebral blood flow in dementia. Arch Neurol. 1975;32:632-637. 1164215 10.1001/archneur.1975.00490510088009

[10] Fischer P Jellinger K Gatterer G Danielczyk W . Prospective neuropathological validation of Hachinski’s Ischaemic Score in dementias. J Neurol Neurosurg Psychiatry. 1991;54:580-583. 1895120 10.1136/jnnp.54.7.580 PMC1014425

[11] Moroney JT Bagiella E Desmond DW , et al. Meta-analysis of the Hachinski Ischemic Score in pathologically verified dementias. Neurology. 1997;49:1096-1105. 9339696 10.1212/wnl.49.4.1096

[12] Molsa PK Paljarvi L Rinne JO Rinne UK Sako E . Validity of clinical diagnosis in dementia: a prospective clinicopathological study. J Neurol Neurosurg Psychiatry. 1985;48:1085-1090. 4078573 10.1136/jnnp.48.11.1085 PMC1028565

[13] Lithgow BJ Dastgheib Z Anssari N , et al. Physiological separation of Alzheimer’s disease and Alzheimer’s disease with significant levels of cerebrovascular symptomology and healthy controls. Med Biol Eng Comput. 2021;59:1597-1610. 34263439 10.1007/s11517-021-02409-8

[14] Roman GC Tatemichi TK Erkinjuntti T , et al. Vascular dementia: Diagnostic criteria for research studies: report of the NINDS-AIREN International Workshop. Neurology. 1993;43:250-250. 8094895 10.1212/wnl.43.2.250

[15] Furtner J Prayer D . Neuroimaging in dementia. Wien Medi Wochenschrift. 2021;171:274-281. 10.1007/s10354-021-00825-x PMC8397686 33660199

[16] McKhann GM Knopman DS Chertkow H , et al. The diagnosis of dementia due to Alzheimer’s disease: recommendations from the National Institute on Aging-Alzheimer’s Association workgroups on diagnostic guidelines for Alzheimer’s disease. Alzheimer’s & Dement. 2011;7:263-269. 10.1016/j.jalz.2011.03.005 PMC3312024 21514250

[17] McConathy J Sheline YI . Imaging biomarkers associated with cognitive decline: A review. Biol Psychiatry. 2015;77:685-692. 25442005 10.1016/j.biopsych.2014.08.024 PMC4362908

[18] Frisoni GB Fox NC Jack CR Scheltens P Thompson PM . The clinical use of structural MRI in Alzheimer disease. Nat Rev Neurol. 2010;6:67-77. 20139996 10.1038/nrneurol.2009.215 PMC2938772

[19] Park M Moon WJ . Structural MR imaging in the diagnosis of Alzheimer’s disease and other neurodegenerative dementia: current imaging approach and future perspectives. Korean J Radiol. 2016;17:827. 27833399 10.3348/kjr.2016.17.6.827 PMC5102911

[20] Baxter LC Sparks DL Johnson SC , et al. Relationship of cognitive measures and gray and white matter in Alzheimer’s disease. J Alzheimer’s Dis. 2006;9:253-260. 16914835 10.3233/jad-2006-9304

[bibr21-26331055231225657] Guo X Wang Z Li K , et al. Voxel-based assessment of gray and white matter volumes in Alzheimer’s disease. Neurosci Lett. 2010;468:146-150. 19879920 10.1016/j.neulet.2009.10.086 PMC2844895

[bibr22-26331055231225657] Li C Du H Zheng J Wang J . A voxel-based morphometric analysis of cerebral gray matter in subcortical ischemic vascular dementia patients and normal aged controls. Int J Med Sci. 2011;8:482-486. 21850200 10.7150/ijms.8.482 PMC3156997

[23] Palesi F De Rinaldis A Vitali P , et al. Specific patterns of white matter alterations help distinguishing Alzheimer’s and vascular dementia. Front Neurosci. 2018;12:274. 29922120 10.3389/fnins.2018.00274 PMC5996902

[24] Tapiola T Alafuzoff I Herukka SK , et al. Cerebrospinal fluid β-amyloid 42 and tau proteins as biomarkers of Alzheimer-type pathologic changes in the brain. Arch Neurol. 2009;66:382-389. 19273758 10.1001/archneurol.2008.596

[25] Schoonenboom NSM Reesink FE Verwey NA , et al. Cerebrospinal fluid markers for differential dementia diagnosis in a large memory clinic cohort. Neurology. 2012;78:47-54. 22170879 10.1212/WNL.0b013e31823ed0f0

[26] Klunk WE Engler H Nordberg A , et al. Imaging brain amyloid in Alzheimer’s disease with Pittsburgh Compound-B. Ann Neurol. 2004;55:306-319. 14991808 10.1002/ana.20009

[27] Jang H Kwon H Yang JJ , et al. Correlations between gray matter and white matter degeneration in pure Alzheimer’s disease, pure subcortical vascular dementia, and mixed dementia. Sci Rep. 2017;7:9541. 28842654 10.1038/s41598-017-10074-x PMC5573310

[28] Kim GH Lee JH Seo SW , et al. Hippocampal volume and shape in pure subcortical vascular dementia. Neurobiol Aging. 2015;36:485-491. 25444608 10.1016/j.neurobiolaging.2014.08.009

[29] Kim HJ Kim J Cho H , et al. Individual subject classification of mixed dementia from pure subcortical vascular dementia based on subcortical shape analysis. PLoS ONE. 2013;8:e75602. 10.1371/journal.pone.0075602 PMC3794958 24130724

[30] Ji F Pasternak O Liu S , et al. Distinct white matter microstructural abnormalities and extracellular water increases relate to cognitive impairment in Alzheimer’s disease with and without cerebrovascular disease. Alzheimers Res Ther. 2017;9:63. 28818116 10.1186/s13195-017-0292-4 PMC5561637

[31] Saha C R. Figley C Dastgheib Z Lithgow B Moussavi Z. A pilot study for investigating differences between Alzheimer’s patients with and without significant vascular pathology. CMBES Proce. 2021;44.

[32] Moussavi Z Koski L Fitzgerald PB , et al. Repeated transcranial magnetic stimulation for improving cognition in Alzheimer disease: Protocol for an interim analysis of a randomized controlled trial. JMIR Res Protoc. 2021;10:1-7. 10.2196/31183 PMC8386362 34383681

[33] Moussavi Z Rutherford G Lithgow B , et al. Repeated transcranial magnetic stimulation for improving cognition in patients with Alzheimer disease: protocol for a randomized, double-blind, placebo-controlled trial. JMIR Res Protoc. 2021;10:12. 10.2196/25144 PMC7822717 33416500

[34] ADNI. Study documents. Accessed February 10, 2022. http://adni.loni.usc.edu/methods/documents/

[35] Gaser C Dahnke R . CAT-A Computational Anatomy Toolbox for the analysis of structural MRI data. HBM. 2016;2016:336-348. 10.1093/gigascience/giae049 PMC11299546 39102518

[36] Manjón JV Coupé P Martí-Bonmatí L Collins DL Robles M . Adaptive non-local means denoising of MR images with spatially varying noise levels. J Magn Reson Imaging. 2010;31:192-203. 20027588 10.1002/jmri.22003

[37] Ashburner J Friston KJ . Unified segmentation. Neuroimage. 2005;26:839-851. 15955494 10.1016/j.neuroimage.2005.02.018

[38] Rajapakse JC Giedd JN Rapoport JL . Statistical approach to segmentation of single-channel cerebral MR images. IEEE Trans Med Imaging. 1997;16:176-186. 9101327 10.1109/42.563663

[39] Ashburner J Friston KJ . Diffeomorphic registration using geodesic shooting and Gauss–Newton optimisation. Neuroimage. 2011;55:954-967. 21216294 10.1016/j.neuroimage.2010.12.049 PMC3221052

[40] Tzourio-Mazoyer N Landeau B Papathanassiou D , et al. Automated anatomical labeling of activations in SPM using a macroscopic anatomical parcellation of the MNI MRI single-subject brain. Neuroimage. 2002;15:273-289. 11771995 10.1006/nimg.2001.0978

[41] Colloby SJ O’Brien JT Taylor JP . Patterns of cerebellar volume loss in dementia with Lewy bodies and Alzheimer’s disease: a VBM-DARTEL study. Psychiatry Res Neuroimaging. 2014;223:187-191. 10.1016/j.pscychresns.2014.06.006 PMC4333903 25037902

[42] Mechelli A Price C Friston K Ashburner J . Voxel-based morphometry of the human brain: methods and applications. Curr Med Imaging Rev. 2005;1:105-113.

[bibr43-26331055231225657] Wang WY Yu JT Liu Y , et al. Voxel-based meta-analysis of grey matter changes in Alzheimer’s disease. Transl Neurodegener. 2015;4:1-9. 25834730 10.1186/s40035-015-0027-z PMC4381413

[44] Zhou X Wu R Zeng Y , et al. Choice of voxel-based morphometry processing pipeline drives variability in the location of neuroanatomical brain markers. Commun Biol. 2022;5:913. 36068295 10.1038/s42003-022-03880-1 PMC9448776

[45] Team RC . R: A language and environment for statistical computing. R Foundation for Statistical Computing. Accessed June 6, 2021. https://www.r-project.org/

[46] Team RS . RStudio: integrated development environment for R. Accessed June 6, 2021. http://www.rstudio.com/

[47] Lieberman MD Cunningham WA . Type I and Type II error concerns in fMRI research: re-balancing the scale. Soc Cogn Affect Neurosci. 2009;4:423-428. 20035017 10.1093/scan/nsp052 PMC2799956

[48] Scheff SW Price DA Schmitt FA Scheff MA Mufson EJ . Synaptic loss in the inferior temporal gyrus in mild cognitive impairment and Alzheimer’s disease. J Alzheimer’s Dis. 2011;24:547-557. 21297265 10.3233/JAD-2011-101782 PMC3098316

[49] Echávarri C Aalten P Uylings HBM , et al. Atrophy in the parahippocampal gyrus as an early biomarker of Alzheimer’s disease. Brain Struct Funct. 2011;3-4:265-271. 10.1007/s00429-010-0283-8 PMC3041901 20957494

[50] Owen AM Milner B Petrides M Evans AC . A specific role for the right parahippocampal gyrus in the retrieval of object-location: a positron emission tomography study. J Cogn Neurosci. 1996;8:588-602. 23961986 10.1162/jocn.1996.8.6.588

[51] Van De Mortel LA Thomas RM Van Wingen GA . Grey matter loss at different stages of cognitive decline: a role for the thalamus in developing Alzheimer’s disease. J Alzheimer’s Dis. 2021;83:705-720. 34366336 10.3233/JAD-210173 PMC8543264

[52] Matsuda H . Voxel-based morphometry of brain MRI in normal aging and Alzheimer’s disease. Aging Dis. 2013;4:29-37. 23423504 PMC3570139

[bibr53-26331055231225657] Wang C Stebbins GT Medina DA , et al. Atrophy and dysfunction of parahippocampal white matter in mild Alzheimer’s disease. Neurobiol Aging. 2012;33:43-52. 20359781 10.1016/j.neurobiolaging.2010.01.020 PMC2910843

[54] Delano-Wood L Stricker NH Sorg SF , et al. Posterior cingulum white matter disruption and its associations with verbal memory and stroke risk in mild cognitive impairment. J Alzheimer’s Dis. 2012;29:589. 22466061 10.3233/JAD-2012-102103 PMC3341099

[55] Roiser JP Linden DE Gorno-Tempini ML Moran RJ Dickerson BC Grafton ST . Minimum statistical standards for submissions to Neuroimage: Clinical. Neuroimage Clin. 2016;12:1045-1047. 27995071 10.1016/j.nicl.2016.08.002 PMC5153601

[56] Martin P Winston GP Bartlett P de Tisi J Duncan JS Focke NK . Voxel-based magnetic resonance image postprocessing in epilepsy. Epilepsia. 2017;58:1653-1664. 28745400 10.1111/epi.13851 PMC5601223

[57] Du AT Schuff N Laakso MP , et al. Effects of subcortical ischemic vascular dementia and AD on entorhinal cortex and hippocampus. Neurology. 2002;58:1635-1641. 12058091 10.1212/wnl.58.11.1635 PMC1820858

[58] van de Pol L Gertz HJ Scheltens P Wolf H . Hippocampal atrophy in subcortical vascular dementia. Neurodegener Dis. 2011;8:465-469. 21613775 10.1159/000326695

[59] de Jong LW van der Hiele K Veer IM , et al. Strongly reduced volumes of putamen and thalamus in Alzheimer’s disease: an MRI study. Brain. 2008;131:3277-3285. 19022861 10.1093/brain/awn278 PMC2639208

[60] Stoodley CJ Valera EM Schmahmann JD . Functional topography of the cerebellum for motor and cognitive tasks: an fMRI study. Neuroimage. 2012;59:1560-1570. 21907811 10.1016/j.neuroimage.2011.08.065 PMC3230671

[61] Childs R Gamage R Münch G Gyengesi E . The effect of aging and chronic microglia activation on the morphology and numbers of the cerebellar Purkinje cells. Neurosci Lett. 2021;751:135807. 33705934 10.1016/j.neulet.2021.135807

[62] Allan LM Ballard CG Rowan EN Kenny RA . Incidence and prediction of falls in dementia: A prospective study in older people. PLoS ONE. 2009;4:e5521. 10.1371/journal.pone.0005521 PMC2677107 19436724

[63] Lee H Wiggermann V Rauscher A , et al. Pilot study of MRI white matter tissue properties in Alzheimer’s, vascular and mixed dementias. Alzheimer’s & Dement. 2020;16:e043961.

[64] Rabinovici GD Seeley WW Kim EJ , et al. Distinct MRI atrophy patterns in autopsy-proven Alzheimer’s disease and frontotemporal lobar degeneration. Am J Alzheimer’s Dise Other Demen. 2008;22:474-488. 10.1177/1533317507308779 PMC2443731 18166607

