# Supplementary material for: Gray and White Matter Voxel-Based Morphometry of Alzheimer’s Disease With and Without Significant Cerebrovascular Pathologies
Source: Neurosci Insights. 2024 Jan 31;19:26331055231225657. doi: 10.1177/26331055231225657 (PMC10832430; doi:10.1177/26331055231225657)
Supplement: sj-docx-1-exn-10.1177_26331055231225657 – Supplemental material for Gray and White Matter Voxel-Based Morphometry of Alzheimer’s Disease With and Without Significant Cerebrovascular Pathologies [file sj-docx-1-exn-10.1177_26331055231225657.docx]

**Supplementary Data:**

Herein, we presented VBM results for comparing AD, AD-CVD and controls after smoothing the modulated GM and WM images by 12 mm FWHM Gaussian kernel.

*Gray Matter VBM:*

Compared to controls, the AD group had significantly lower GM volume in several brain regions after correcting for age, sex, TIV, and MRI location (Table S1 and Figure S1 A-C). The largest of these clusters (236332 voxels) was primarily located in the right inferior temporal gyrus and parahippocampal gyrus, but also extended to other brain areas. The second and third largest clusters (5540 and 5175 voxels) were mostly centered in the right middle and superior temporal gyrus and the dorsolateral part of the right superior frontal gyrus, respectively. However, not all differences were right-lateralized; a large cluster of 3109 voxels was observed in the left thalamus. Other significant clusters were also found in the opercular part of the right inferior frontal gyrus and right precentral gyrus, right angular gyrus and middle occipital gyrus, right inferior parietal gyrus, right thalamus, left inferior temporal gyrus, right supramarginal gyrus, left angular gyrus, left middle temporal gyrus, and right Rolandic operculum. No substantial differences were found for the opposite t-contrast (AD > controls).

**Table S1:** Regions of lower gray matter (GM) volume in AD and AD-CVD compared with controls

| **Voxel level**  ***P*_FWE-corr_** | **Extent** | **T values of voxel-level** | **Peak MNI coordinates**  **(x, y, z) (mm)** | **Side**  **(L: Left,**  **R: Right)** | **Regions** |
| --- | --- | --- | --- | --- | --- |
| **AD < controls** | | | | | |
| < 0.001 | 236332 | 13.93 | 48, -42, -24 | R | Inferior temporal gyrus |
| < 0.001 |  | 13.49 | 20, -12, -28 | R | Parahippocampal gyrus |
| < 0.001 |  | 13.49 | 26, -20, -26 | R | Parahippocampal gyrus |
| < 0.001 | 23 | 12.20 | -54, -44, -22 | L | Inferior temporal gyrus |
| < 0.001 | 3109 | 9.39 | -2, -6, 4 | L | Thalamus |
| < 0.001 | 5540 | 9.09 | 64, -30, 0 | R | Middle temporal gyrus |
| < 0.001 |  | 7.60 | 60, -28, 20 | R | Superior temporal gyrus |
| < 0.001 |  | 7.46 | 60, -44, 12 | R | Superior temporal gyrus |
| < 0.001 | 1140 | 7.64 | 50, 10, 28 | R | Inferior frontal gyrus, opercular part |
| < 0.001 |  | 5.60 | 46, 2, 44 | R | Precentral gyrus |
| < 0.001 | 5175 | 7.36 | 24, 14, 56 | R | Superior frontal gyrus, dorsolateral |
| < 0.001 |  | 7.31 | 22, 30, 48 | R | Superior frontal gyrus, dorsolateral |
| < 0.001 |  | 7.10 | 24, 24, 52 | R | Superior frontal gyrus, dorsolateral |
| < 0.001 | 22 | 7.24 | 58, -26, 42 | R | Supramarginal gyrus |
| < 0.001 | 95 | 7.01 | 46, -40, 50 | R | Inferior parietal gyrus |
| < 0.001 | 2 | 6.98 | -52, 4, -26 | L | Middle temporal gyrus |
| < 0.001 | 259 | 6.48 | 34, -66, 42 | R | Angular gyrus |
| 0.003 |  | 4.84 | 32, -74, 36 | R | Middle occipital gyrus |
| < 0.001 | 89 | 5.90 | 16, -32, 2 | R | Thalamus |
| < 0.001 | 2 | 5.57 | 60, -12, 14 | R | Rolandic operculum |
| < 0.001 | 8 | 5.35 | -42, -66, 40 | L | Angular gyrus |
| 0.038 | 1 | 4.17 | 16, -10, 68 | R | Superior frontal gyrus, dorsolateral |
| **AD-CVD < controls** | | | | | |
| < 0.001 | 228176 | 12.49 | 48, -44, -24 | R | Inferior temporal gyrus |
| < 0.001 |  | 11.69 | 50, -54, -22 | R | Inferior temporal gyrus |
| < 0.001 |  | 11.47 | 20, -8, -30 | R | Parahippocampal gyrus |
| < 0.001 | 23 | 10.22 | -54, -44, -22 | L | Inferior temporal gyrus |
| < 0.001 | 5539 | 8.48 | 64, -30, -2 | R | Middle temporal gyrus |
| < 0.001 |  | 7.50 | 62, -28, 18 | R | Superior temporal gyrus |
| < 0.001 |  | 7.11 | 40, -20, 12 | R | Heschl gyrus |
| < 0.001 | 2506 | 8.01 | -2, -8, 2 | L | Thalamus |
| < 0.001 |  | 7.44 | -4, -14, 8 | L | Thalamus |
| < 0.001 | 932 | 7.08 | 50, 10, 28 | R | Inferior frontal gyrus, opercular part |
| < 0.001 |  | 5.36 | 48, 2, 42 | R | Precentral gyrus |
| < 0.001 | 403 | 6.49 | -46, -40, 48 | L | Inferior parietal gyrus |
| < 0.001 |  | 4.86 | -52, -38, 42 | L | Inferior parietal gyrus |
| < 0.001 | 3525 | 6.17 | 22, 36, 40 | R | Superior frontal gyrus, dorsolateral |
| < 0.001 |  | 5.94 | 30, 50, 22 | R | Middle frontal gyrus |
| < 0.001 |  | 5.88 | 24, 18, 54 | R | Middle frontal gyrus |
| < 0.001 | 2 | 6.03 | 60, -12, 14 | R | Rolandic operculum |
| < 0.001 | 2 | 6.00 | -52, 4, -26 | L | Middle temporal gyrus |
| < 0.001 | 22 | 5.86 | 58, -28, 40 | R | Supramarginal gyrus |
| < 0.001 | 95 | 5.78 | 46, -40, 50 | R | Inferior parietal gyrus |
| 0.018 | 66 | 4.37 | 34, -66, 42 | R | Angular gyrus |
| 0.039 |  | 4.16 | 26, -68, 46 | R | Superior occipital gyrus |
| 0.018 | 8 | 4.37 | -44, -66, 40 | L | Angular gyrus |
| 0.037 | 1 | 4.18 | 16, -32, 2 | R | Thalamus |

***Note:*** *P_FWE-corr_ = Family-wise error (FWE) corrected p-values, MNI = Montreal Neurological Institute.*

The AD-CVD individuals also had substantially lower GM volume in brain regions relative to the control group, as described in Table S1 and Figure S1 D-F. Similar to the AD group, the largest of these clusters (228176 voxels) was primarily positioned in the right inferior temporal gyrus and parahippocampal gyrus and covered several brain regions of AD-CVD. The second-largest cluster of 5539 voxels was mainly centered in the right middle and superior temporal gyrus and right Heschl gyrus. Large clusters of GM differences in AD-CVD compared to controls were also found in regions of the dorsolateral part of the right superior frontal gyrus and right middle frontal gyrus, left thalamus, opercular part of the right inferior frontal gyrus and right precentral gyrus and left inferior parietal gyrus. Other clusters with < 100 voxels were in the right inferior parietal gyrus, right angular gyrus, and superior occipital gyrus, left inferior temporal gyrus, right supramarginal gyrus, left angular gyrus, left middle temporal gyrus, right Rolandic operculum, and right thalamus. No significant differences were noticed for the opposite t-contrast (AD-CVD > controls).


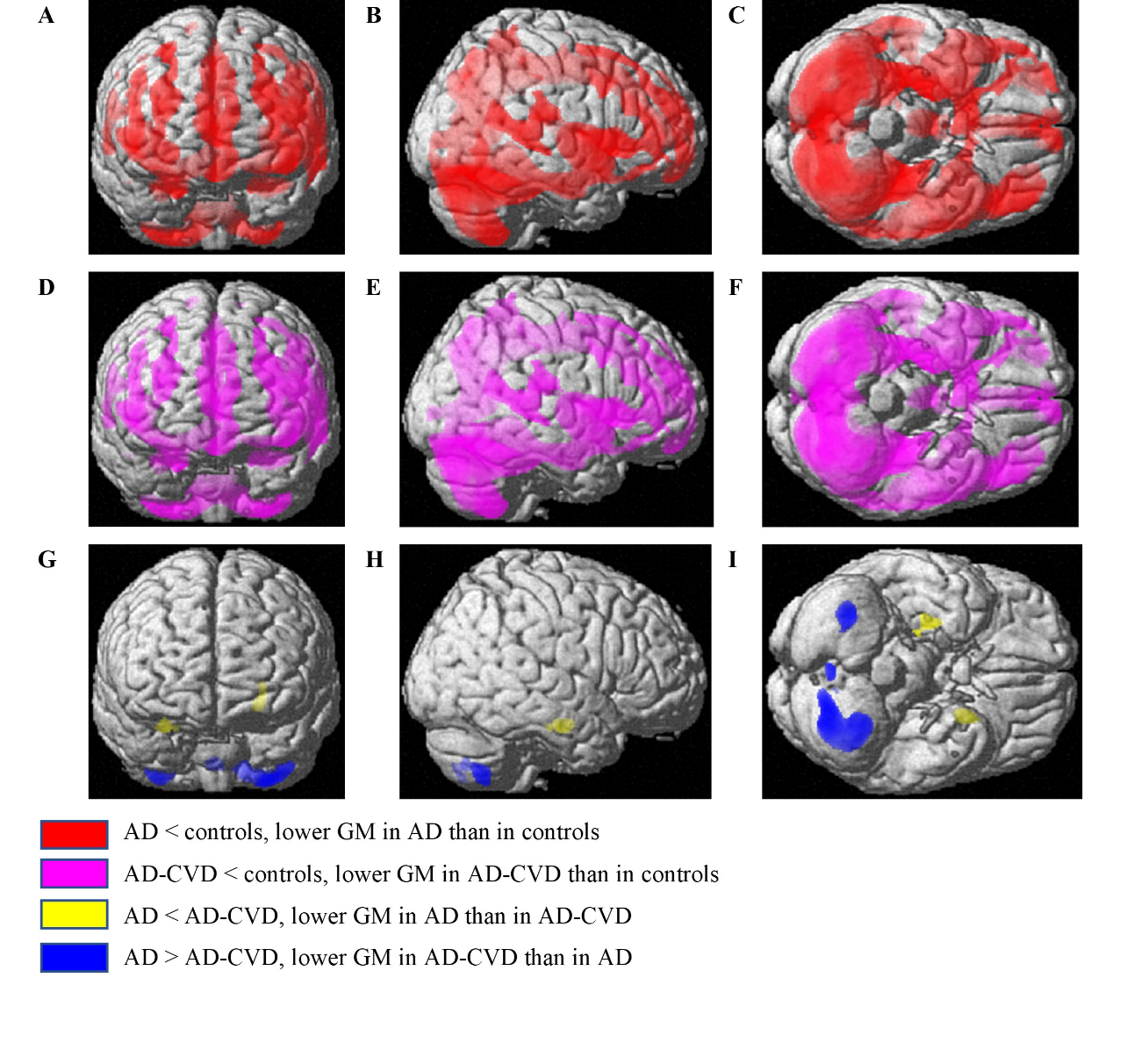


**Figure S1:** Anatomical rendering in SPM displays voxel-based morphometry results on gray matter (GM). The left, middle, and right columns are anterior, right, and bottom views of the 3D brain template, respectively. In the t-contrasts of AD < controls [A-C] and AD-CVD < controls [D-F], the family-wise error (FWE) was corrected at p = 0.05; an uncorrected p < 0.005 with K = 10 ^44^ was used for the AD < AD-CVD and AD > AD-CVD contrasts [G-I]. Both dementia groups demonstrated widespread patterns of significantly lower GM volume than controls, and the GM volumetric difference patterns of AD and AD-CVD compared to controls appeared to be overlapped. In comparison between the two dementia groups, AD showed a trend of lower GM, particularly in the right hippocampus, right parahippocampal gyrus, and left putamen compared to AD-CVD. Relative to AD, the AD-CVD had lower GM, mainly in the lobules of the cerebellum and vermis. AD: Alzheimer’s Disease, AD-CVD: AD with cerebrovascular disease.

Amongst the contrasts between the two dementia groups (AD < AD-CVD, AD > AD-CVD), a significant difference was not observed in VBM analysis using P_FWE-corr_ < 0.05. However, using an uncorrected threshold of p < 0.005 with K = 10, as presented in Table S2 and Figure S1 G-I, AD subjects exhibited a trend of lower GM volume in the right hippocampus and parahippocampal gyrus and left putamen compared to AD-CVD. In the opposite contrast of AD > AD-CVD, AD-CVD exhibited a trend of lower GM volume in the lobule 7b (left) and 8 (left, right) of cerebellar hemisphere, lobule 8 of vermis, and lingual gyrus relative to AD.

**Table S2:** Regions of gray matter (GM) and white matter (WM) difference in AD compared to AD-CVD

| **t-contrast** | **Extent** | **T values of voxel-level** | **Peak MNI coordinates**  **(x, y, z) (mm)** | **Side**  **(L: Left,**  **R: Right)** | **Regions** |
| --- | --- | --- | --- | --- | --- |
| **GM** | | | | | |
| AD < AD-CVD | 460 | 3.12 | 32, -10, -24 | R | Hippocampus |
|  |  | 2.71 | 24, -20, -24 | R | Parahippocampal gyrus |
|  | 609 | 2.90 | -24, 6, -10 | L | Putamen |
|  |  | 2.71 | -26, 14, -2 | L | Putamen |
| AD > AD-CVD | 4772 | 4.05 | -28, -76, -52 | L | Cerebellum (7b) |
|  |  | 3.33 | -32, -52, -58 | L | Cerebellum (8) |
|  |  | 3.04 | -42, -58, -50 | L | Cerebellum (7b) |
|  | 1039 | 3.33 | 30, -62, -48 | R | Cerebellum (8) |
|  | 275 | 2.91 | 2, -70, -46 | R | Vermis (8) |
|  | 10 | 2.63 | -10, -66, -8 | L | Lingual gyrus |
| **WM** | | | | | |
| AD < AD-CVD | 49 | 2.88 | 16, -14, 18 | R | Thalamus |
|  | 25 | 2.76 | 36, 48, 0 | R | Middle frontal gyrus |
| AD > AD-CVD | 640 | 2.92 | 2, -36, -30 | R | Brainstem |

***Note:****Results are presented here at a liberal threshold of uncorrected p < 0.005 and K = 10. AD and AD-CVD groups showed a tendency of GM and WM differences in the regions mentioned above while comparing them to each other. MNI = Montreal Neurological Institute.*

## *White Matter VBM*

Relative to the control group, AD individuals were found to have lower WM volume in several regions after correcting for age, sex, TIV, and MRI location (Table S3 and Figure S2 A-C). These were mainly located in the left hippocampus, left insula, and orbital part of the left inferior frontal gyrus, with the largest cluster of 183146 voxels. Moreover, the cerebellar lingual, left culmen and right cerebellum (3) presented a substantially lower WM in AD than in controls. No substantial differences were found for the opposite t-contrast (AD > controls).


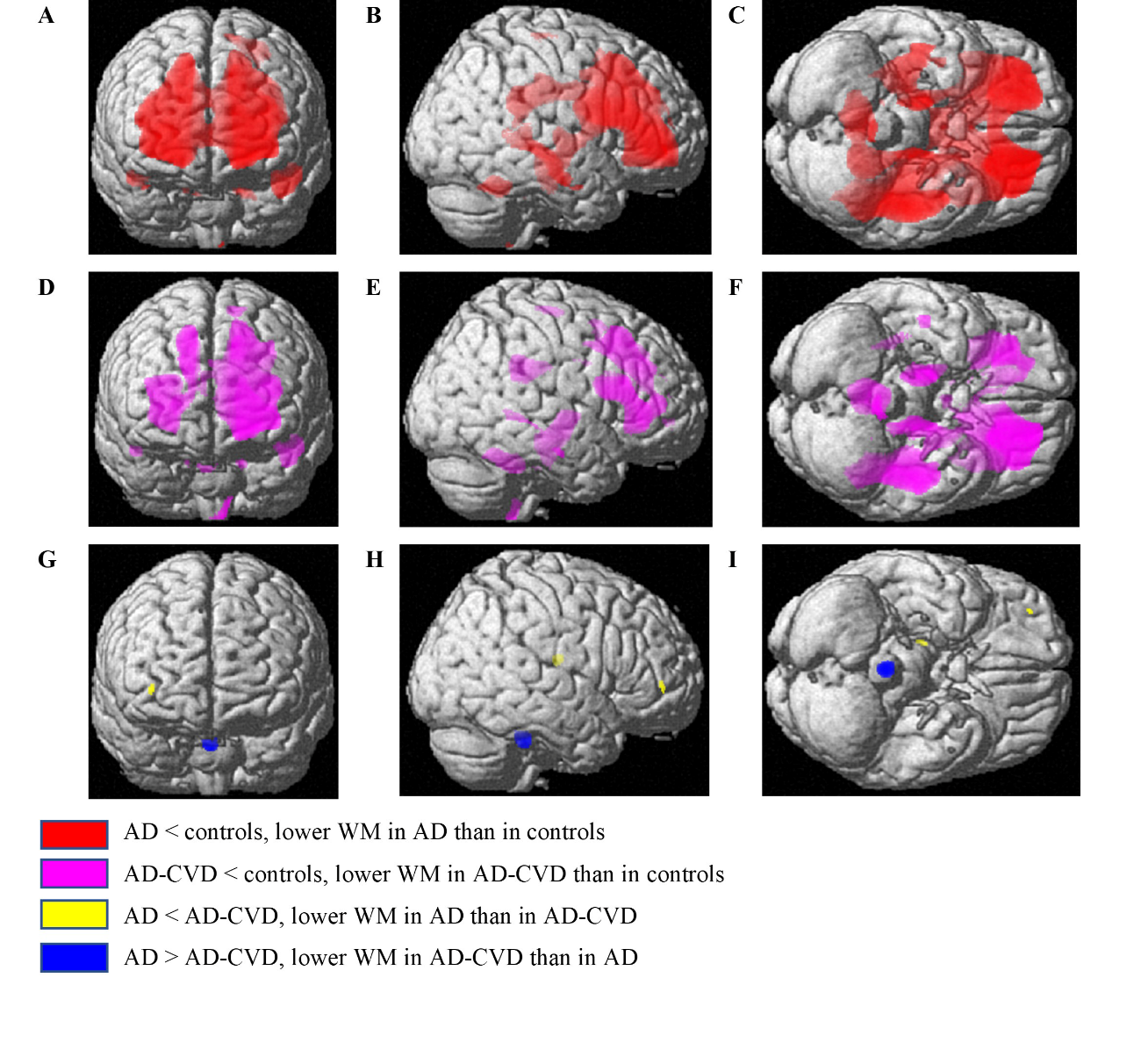


**Figure S2:** Anatomical rendering in SPM displays voxel-based morphometry results on white matter (WM). The left, middle, and right columns are anterior, right, and bottom views of the 3D brain template, respectively. In the t-contrasts of AD < controls [A-C] and AD-CVD < controls [D-F], the family-wise error (FWE) was corrected at p = 0.05 and an uncorrected thresholding of p < 0.005 with K = 10 was used for the AD < AD-CVD and AD > AD-CVD contrasts [G-I]. Compared to controls, AD and AD-CVD groups presented a significantly lower WM volume in diverse brain areas, and most of the different regions appeared to be overlapped in both disease states. While comparing the two dementia groups, there was a subtle trend of WM difference in colored regions between AD and AD-CVD. AD = Alzheimer’s disease, AD-CVD = AD with cerebrovascular disease

**Table S3:** Regions of lower white matter (WM) volume in AD and AD-CVD groups compared with controls.

| **Voxel level**  ***P*_FWE-corr_** | **Extent** | **T values of voxel-level** | **Peak MNI coordinates**  **(x, y, z) (mm)** | **Side**  **(L: Left,**  **R: Right)** | **Regions** |
| --- | --- | --- | --- | --- | --- |
| **AD < controls** | | | | | |
| < 0.001 | 183146 | 6.77 | -34, -24, -10 | L | Hippocampus |
| < 0.001 |  | 6.74 | -30, 20, 14 | L | Insula |
| < 0.001 |  | 6.45 | -24, 32, -12 | L | Inferior frontal gyrus, orbital part |
| 0.015 | 138 | 4.27 | 0, -44, -66 | - | Cerebellar Lingual |
| 0.034 |  | 4.02 | -6, -46, -60 | L | Culmen |
| 0.046 | 2 | 3.93 | 18, -24, -26 | R | Cerebellum (3) |
| **AD-CVD < controls** | | | | | |
| < 0.001 | 72764 | 5.87 | -14, 22, 46 | L | Superior frontal gyrus, dorsolateral |
| < 0.001 |  | 5.73 | -20, 20, -4 | L | Putamen |
| < 0.001 |  | 5.51 | -36, -22, -12 | L | Hippocampus |
| 0.001 | 2867 | 5.07 | 4, -46, -24 | R | Vermis (1, 2) |
| 0.001 |  | 4.90 | 4, -54, -18 | R | Vermis (4, 5) |
| 0.002 | 3031 | 4.77 | 16, -18, -14 | R | Thalamus |
| 0.026 |  | 4.11 | 16, -20, -26 | R | Pons |
| 0.003 | 525 | 4.69 | -12, -20, 66 | L | Paracentral lobule |
| 0.004 | 6153 | 4.65 | -8, -28, 28 | L | Median cingulate & paracingulate gyri |
| 0.016 |  | 4.25 | -18, -44, 38 | L | Cingulate gyrus |
| 0.033 |  | 4.04 | -14, -28, 38 | L | Cingulate gyrus |
| 0.007 | 135 | 4.08 | 34, -38, -2 | R | Hippocampus |
| 0.037 |  | 4.00 | 36, -30, -4 | R | Sub-lobar |
| 0.008 | 2253 | 4.45 | 12, -38, 26 | R | Median cingulate & paracingulate gyri |
| 0.042 |  | 3.97 | 8, -20, 30 | R | Cingulate gyrus |
| 0.016 | 137 | 4.25 | 50, -16, -22 | R | Inferior temporal gyrus |
| 0.018 | 398 | 4.21 | -6, -44, -62 | L | Cerebellum (9) |
| 0.034 |  | 4.02 | -10, -42, -50 | L | Cerebellum (9) |
| 0.035 | 2 | 4.02 | 30, -46, 4 | R | Sub-lobar |
| 0.040 | 12 | 3.98 | 0, -4, 26 | - | Corpus callosum |
| 0.044 | 2 | 3.95 | 38, -26, -8 | R | Hippocampus |
| 0.047 | 1 | 3.93 | 38, -24, -10 | R | Hippocampus |
| 0.047 | 1 | 3.93 | -22, -24, -10 | L | Hippocampus |
| 0.047 | 1 | 3.93 | -22, -46, -26 | L | ver(4, 5) |
| 0.048 | 1 | 3.93 | 40, -26, -8 | R | Sub-gyral |
| 0.048 | 1 | 3.92 | 16, -20, -30 | R | Pons |
| 0.049 | 1 | 3.92 | 8, 28, 20 | R | Anterior cingulate & paracingulate gyri |
| 0.049 | 2 | 3.92 | 4, -4, 26 | R | Sub-lobar |
| 0.049 | 3 | 3.92 | -16, 42, 30 | L | Superior frontal gyrus, dorsolateral |
| 0.049 | 1 | 3.91 | -14, -24, 42 | L | Median cingulate & paracingulate gyri |

***Note:*** *P_FWE-corr_ = Family-wise error (FWE) corrected p-values, MNI = Montreal Neurological Institute.*

Comparing the AD-CVD and control groups (Table S3 and Figure S2 D-F) revealed that the AD-CVD group showed substantially lower WM volumes in several regions, particularly in the left superior frontal gyrus (dorsolateral part), putamen, and hippocampus, with the largest cluster of 72764 voxels. The second largest cluster (6153 voxels) was demonstrated in regions of the left cingulate gyrus. However, not all differences were left-lateralized; the surrounding of the thalamus, vermis and cingulate gyrus on the right side indicated substantially lower WM in AD than in controls. Besides, relative to controls, the AD-CVD group demonstrated a lower WM in the left paracentral lobule, lobule 9 (left) of cerebellum, right inferior temporal gyrus, and right hippocampus. No significant differences were noticed for the opposite t-contrast (AD-CVD > controls).

In comparing AD and AD-CVD using the uncorrected thresholding of p < 0.005 and K = 10, as shown in Table S2 and Figure S2 G-I, AD tended to exhibit lower WM volumes in right thalamus and middle frontal gyrus than AD-CVD. The AD-CVD relative to AD appeared to demonstrate lower WM, mainly in regions of the right brainstem.
